# Genome-wide identification of WRKY genes and their expression profiles under different abiotic stresses in *Elaeis guineensis*

**DOI:** 10.1371/journal.pone.0189224

**Published:** 2017-12-11

**Authors:** Yong Xiao, Lixia Zhou, Xintao Lei, Hongxing Cao, Yong Wang, Yajing Dou, Wenqi Tang, Wei Xia

**Affiliations:** 1 Coconuts Research Institute, Chinese Academy of Tropical Agricultural Sciences, Wenchang, P.R. China; 2 Hainan Key Laboratory for Sustainable Utilization of Tropical Bioresources, Institute of Tropical Agriculture and Forestry, Hainan University, Haikou, P.R China; Huazhong University of Science and Technology, CHINA

## Abstract

African oil palm (*Elaeis guineensis*) is an important oil crop grown in tropical region and sensitive to low temperature along with high tolerance to salt and drought stresses. Since the *WRKY* transcription factor family plays central roles in the regulation of plant stress tolerance, 95 genes belonging to the *WRKY* family were identified and characterized in oil palm genome. Gene structure analysis showed that *EgWRKY* genes have considerable variation in intron number (0 to 12) and gene length (477bp to 89,167 bp). Duplicated genes identification indicated 32 *EgWRKY* genes originated from segmental duplication and two from tandem duplication. Based on transcriptome data, most *EgWRKY* genes showed tissue-specific expression patterns and their expression could be induced under cold stress. Furthermore, six *EgWRKY* genes with more than two-folded increased expression level under cold stress were validated by RT-qPCR, which has higher expression level in cold, drought and high salinity treatment. The identification and characterization of WRKY gene family showed that *EgWRKY* were associated with a wide range of abiotic stress responses in *Elaeis guineensis* and some *EgWRKY* members with high expression levels could be selected for further research in analyzing their functions in the stress response in African oil palm.

## Introduction

African oil palm (2n = 32, *Elaeis guineensis*), belonging to the family Arecaceae, is a major tropical oil crop worldwide, which has the highest oil production per unit area. Presently, this tropical crop is cultivated in Southeast Asia, Africa, Central America and Brazil. In 2012, the total yield of African oil palm was approximately 50 million tons of palm oil from 17 million hectares of plantation (http://faostat3.fao.org/home/E). The whole-genome sequence of *Elaeis guineensis* was completed and released in2013 and a total of 1.53 Gb of sequence data was assembled with 34,802 genes annotated [1]. As a tropical oil crop, *Elaeis guineensis* is particularly sensitive to low temperature but has high tolerance to salt and drought stresses. Identifying and validating genes related with stress response processes in African oil palm especially for cold responsive process will assist the further molecular breeding of stress tolerance cultivars.

*WRKY* genes have been identified and functionally analysed in various species. A large proportion of *WRKY* genes were found to be involved in various biotic [2–7] and abiotic stresses [8–14]. The expression of *WRKY* gene can also be induced by abscisic acid [15], gibberellins (GA) [16], salicylic acid [17], jasmonic acid [18], and ethylene treatment [19]. In Arabidopsis, the expression of *AtWRKY*30can be induced by abiotic stress and methyl viologen (MV), H_2_O_2_, arsenic, drought, NaCl, and mannitol treatment. Over-expression of*AtWRKY*30can significantly increase the tolerance of Arabidopsis to MV and salinity stress [20]. Meanwhile, some WRKYs in rice were also found to be associated with abiotic stress, including *OsWRKY*11 [11], *OsWRKY*30[14], *OsWRKY*45[21], and *OsWRKY*47 [22]. Mutation of *WRKY*46 can decrease the tolerance to osmotic and salt stress in Arabidopsis [23]. The expression levels of *WRKY*25 and *WRKY*26 can be up-regulated by heat stress [24]. Moreover, in Arabidopsis, *WRKY*53 was validated in negative regulation of leaf senescence [25]. Some *WRKY* proteins were also shown to be phosphorylated by MAPKs and to contain a conserved motif for this phosphorylation [14]. Identification and characterization of WRKY genes in African oil palm could show a clue for genes involved in its stress responsive processes.

The *WRKY* transcription factors generally contain one or two conserved *WRKY* domains and a zinc finger motif and could be divided into three large clusters based on the variations in *WRKY* and zinc finger motifs [26]. Members of cluster I contain two conserved *WRKY* motifs with a C_2_H_2_ motif. ClustersII and III are characterized by a single conserved *WRKY* motif. The functional amino acid sequence of a *WRKY* motif is WRKYGQK and existed different variations, including WRKYGKK, WRKYGEK, WRKYGRK, WKKYGQK, WKRYGQK, and WSKYEQK. Meanwhile, there are two types of zinc finger motifs in *WRKY* transcription factors: the C_2_H_2_ motif (C-X_4-5_-C-X_22-23_-H-X_1_-H) and the C_2_HC motif (C-X_5-7_-C-X_2-3_-H-X_1_-C) [26].

To date, many *WRKY* genes in different species have been identified, including 74 *WRKY* genes in Arabidopsis [27], 103 in rice [28], 45 in barley [29], 55 in cucumber [30], 119 in maize [31], 182 in soybean [32], 109 in cotton [33], and 85 in cassava [34]. However, there is still no systematic identification and characterization of *WRKY*s conducted in African oil palm to find a clue for candidate WRKY genes involved in the stress responsive processes. In this study, genome-wide scanning for WRKY genes in African oil palm were conducted based on its genome sequence and the gene structures, phylogenetic relationships, expression profiles for all WRKY genes were also been performed. This research will provide insight into the functionality of *EgWRKY*s in this tropical oil crop.

## Plant materials and methods

### Plant materials and stress treatments

The seedlings of African oil palm (*pisifera*, thin-shelled) were grown in nurseries before stress treatments. Thirty-six oil palm plants germinated in the same week and grown in the same nursery were selected for subsequent cold, drought and salt treatment. The control seedlings for the three kinds of treatment were grown under 16 hours light/8 hours dark photoperiod at 26°C and three biological replicates were set in each stress treatment. For the cold treatment, a set of 12 seedling was placed in a growth chamber at 26°C for one day. Three oil palm plants were used as controls and the rest nine oil palm plants were kept at 8°C for 4, 24, and 48 hours under 16 hours light/8 hours dark photoperiod. Spear leaves were sampled from control and cold-treated seedlings and immediately frozen in liquid nitrogen. For the drought treatment, when soil water content reached 23% (2.3 g water per 10 g soil), spear leaves were sampled after 0, 4, 24 and 48 hours and immediately frozen in liquid nitrogen. For the salt treatment, the roots of oil palm seedlings were soaked in a NaCl solution (400 mmol/L)under 16 hours light/8 hours dark photoperiod at 26°C, and spear leaves were sampled 0, 4, 24, and 48 hours after salt treatment and immediately frozen in liquid nitrogen for RNA isolation. The MRIP method was used to extract RNA from these leaves [35].

### Identification and phylogenetic analysis of *EgWRKY*s

The whole-genome sequence of *Elaeis guineensis* was downloaded from the National Center for Biotechnology Information (NCBI). Sequences of *AtWRKY* genes were downloaded from the Arabidopsis Information Resource (TAIR), available at http://www.arabidopsis.org. To identify *EgWRKY*s in *Elaeis guineensis*, *AtWRKY* sequences were used as queries to blast against all gene models in African oil palm, using a cutoff E-value of 1e^-10^. Multiple protein sequences alignment was used to confirm the conserved domains of *EgWRKY* sequences. Meanwhile, the conserved motifs were also predicted by alignment with CDD (http://www.ncbi.nlm.nih.gov/cdd) and PFAM databases (http://pfam.sanger.ac.uk). The MEME program (http://meme-suite.org) was used to identify the conserved motifs of E*gWRKY*s. MEGA 5.0 software was used to construct a neighbour-joining phylogenetic tree based on amino acid sequences of conserved *WRKY* domain with 1000 bootstrap replicates [36, 37].

### Characterization of gene structures and gene duplication events of *EgWRKY*

The gene annotation summaries, CDSs, and mRNA sequences of *Elaeis guineensis* was downloaded from NCBI. Gene structures of *EgWRKY*s in *Elaeis guineensis* were confirmed by aligning mRNA sequences with the whole-genome sequence of *Elaeis guineensis*. The gene structures were identified by the Gene Structure Display Server programme. The duplication events of all *EgWRKY* identified in this study were analysed using an algorithm that can scan multiple genomes or subgenomes to identify putative homologous chromosomal regions and align these regions using genes as anchors [38].

### The expression profiles analysis of *EgWRKY*s based on transcriptome datasets

The raw data of nine transcriptomes from different tissues, including mesocarp (five different developmental stages), leaf, fruit, flower, root, and shoot, were downloaded from the SRA (Sequence Read Archive) database of the NCBI website. RPKM (reads per kb per million reads)values were used to calculate gene expression levels using the following formula[39]:

RPKM=106CNL103

where C is the number of reads that aligned exclusively with one expressed sequence; N is the total number of reads that aligned with all expressed sequences; and L is the number of bases in the CDS of the corresponding sequence.

### Real-time qPCR assays

Quantitative real-time PCR was carried out using a standard SYBR Premix Ex Taq^™^ kit (TaKaRa) protocol in 96-well optical plates (Axygen) with a final reaction volume of 10 μl. The real-time qPCRs were incubated in 0.2-ml tubes in a Mastercycler ep realplex4 (Eppendorf) machine as follows: 95°C for 5 seconds, 55°C for 15 seconds and 68°C for 20 seconds. The procedure ended with a melt curve ramping from 60 to 95°C for 20 minutes to verify the PCR specificity. All qPCRs were carried out in biological and technical triplicate. The final Ct values were the means of nine measurements. The expression levels of the selected *EgWRKY*s were normalized to those of ELF, which was previously found to be a stable reference gene under abiotic stress [40]. One-way ANOVA was used to test significant difference (LSD, *p*< 0.05) of expression level between different time periods after cold, drought and salt treatment. All expression data was analyzed using SPSS software.

## Results

### Identification of *EgWRKY* transcription factors in the genome of *Elaeis guineensis*

The identification of *EgWRKY* genes in African oil palm were using the amino acid sequences of 72 *AtWRKY* genes were used as query sequences with a cutoff E-value < 10^-5^via BLAST analysis. The blast results showed that 97orthologous genes of *AtWRKY* were found in home-made database containing all protein coding genes from African oil palm and these genes could be classified into the *EgWRKY* gene family. The conserved WRKY domain sequences analysis in these *EgWRKY* genes excluded two genes from further analysis due to a lack of the conserved *WRKY* domain. These remaining *EgWRKY*s were designated from *EgWRKY*01 to *EgWRKY*95 according to their genomic location in African oil palm. Detailed information about the 95 *EgWRKY* genes was listed in Table 1 (S1 and S2 Text).

**Table 1:** Detailed information for the *EgWRKY* genes identified in the genome of *Elaeis guineensis*.

Gene Symbol	Chromosome No.	Gene LOC	Strand	Start	End	Peptide Length
WRKY01	1	LOC105049863	minus	2828118	2834829	237
WRKY02	1	LOC105054716	plus	3528352	3531492	517
WRKY03	1	LOC105055190	minus	3581086	3582588	196
WRKY04	1	LOC105055340	plus	3587901	3589580	206
WRKY05	1	LOC105038749	minus	3597496	3599292	202
WRKY06	1	LOC105056374	minus	3790630	3791759	176
WRKY07	1	LOC105039486	minus	8508520	8510767	356
WRKY08	1	LOC105042030	minus	10329250	10329726	158
WRKY09	1	LOC105041582	minus	10334215	10335866	346
WRKY10	1	LOC105041458	plus	10559780	10561375	276
WRKY11	1	LOC105049072	plus	18267529	18269693	342
WRKY12	1	LOC105048544	plus	19074456	19077025	588
WRKY13	1	LOC105058356	minus	45150214	45153149	258
WRKY14	1	LOC105058306	plus	46090220	46106758	947
WRKY15	2	LOC105035500	plus	1244849	1249774	500
WRKY16	2	LOC105038142	minus	18378213	18380205	304
WRKY17	2	LOC105038817	minus	34420487	34423323	181
WRKY18	2	LOC105038993	minus	38960111	38968368	738
WRKY19	2	LOC105039302	plus	47764368	47766219	316
WRKY20	2	LOC105039542	minus	49916408	49921049	739
WRKY21	2	LOC105039938	plus	60595545	60597170	254
WRKY22	3	LOC105040295	minus	2897890	2903252	240
WRKY23	3	LOC105040365	plus	3976191	3981682	632
WRKY24	3	LOC105040375	minus	4421975	4423228	208
WRKY25	3	LOC105040376	plus	4455296	4457347	171
WRKY26	3	LOC105040720	plus	11542191	11545474	373
WRKY27	3	LOC105040793	minus	12905634	12907531	374
WRKY28	3	LOC105041017	plus	16840840	16843289	532
WRKY29	3	LOC105042138	plus	43201571	43208037	186
WRKY30	3	LOC105042164	plus	46869949	46873630	358
WRKY31	4	LOC105043766	plus	46688084	46692604	213
WRKY32	4	LOC105043986	plus	50354451	50358395	419
WRKY33	5	LOC105044378	plus	1003267	1006117	326
WRKY34	5	LOC105044758	minus	5971453	5974564	594
WRKY35	5	LOC105044827	minus	7468889	7471135	291
WRKY36	5	LOC105044828	minus	7509255	7511846	276
WRKY37	5	LOC105045089	minus	7516052	7519209	210
WRKY38	5	LOC105045090	minus	7533908	7536374	365
WRKY39	5	LOC105044992	minus	11466843	11468463	311
WRKY40	5	LOC105045992	plus	42769327	42771373	310
WRKY41	5	LOC105045991	plus	42856852	42860147	323
WRKY42	5	LOC105046191	plus	44348667	44361739	1303
WRKY43	5	LOC105045782	plus	47101333	47106104	469
WRKY44	5	LOC105045639	minus	48908668	48911631	314
WRKY45	6	LOC105046551	minus	3040168	3043302	356
WRKY46	6	LOC105046621	minus	4288074	4293918	462
WRKY47	6	LOC105046637	plus	4774207	4779820	302
WRKY48	6	LOC105047221	minus	33625556	33627842	575
WRKY49	6	LOC105047232	minus	34241286	34243364	336
WRKY50	6	LOC105047488	minus	38804476	38806170	277
WRKY51	6	LOC105047514	plus	39157107	39158687	328
WRKY52	6	LOC105047564	minus	40484853	40487251	360
WRKY53	6	LOC105047700	minus	42604078	42605690	202
WRKY54	6	LOC105047725	plus	43055219	43060457	235
WRKY55	7	LOC105047930	plus	2603710	2610166	626
WRKY56	7	LOC105047934	minus	2664908	2666305	210
WRKY57	7	LOC105047936	plus	2698084	2699488	166
WRKY58	7	LOC105048100	minus	5565923	5567535	287
WRKY59	7	LOC105048220	plus	7728041	7731472	369
WRKY60	7	LOC105048265	minus	8642979	8644669	371
WRKY61	7	LOC105048552	plus	10622308	10624799	584
WRKY62	7	LOC105049007	plus	26175565	26204615	170
WRKY63	7	LOC105049350	plus	40437708	40439232	324
WRKY64	8	LOC105050028	minus	23543798	23554103	742
WRKY65	8	LOC105050398	plus	31318423	31319807	308
WRKY66	8	LOC105050724	minus	36521373	36525318	257
WRKY67	8	LOC105050725	minus	36538138	36539837	315
WRKY68	8	LOC105050730	plus	36672320	36680988	533
WRKY69	8	LOC105050834	plus	38477347	38479734	321
WRKY70	9	LOC105051963	plus	33121964	33123639	292
WRKY71	10	LOC105052206	minus	2919960	3009126	514
WRKY72	10	LOC105052255	minus	5488658	5491941	530
WRKY73	10	LOC105052308	minus	7533000	7535705	312
WRKY74	10	LOC105052410	minus	7555119	7557529	243
WRKY75	10	LOC105052589	plus	16498194	16500066	283
WRKY76	10	LOC105053348	minus	27936570	27938051	116
WRKY77	10	LOC105053433	plus	30816717	30820483	353
WRKY78	11	LOC105053799	plus	25944218	25948814	421
WRKY79	14	LOC105056919	minus	1936977	1939615	336
WRKY80	14	LOC105057172	minus	3999586	4002656	596
WRKY81	14	LOC105057372	minus	4789612	4792391	385
WRKY82	14	LOC105057329	minus	6594036	6597123	315
WRKY83	15	LOC105058006	minus	7368664	7381113	551
WRKY84	-	LOC105037454	plus	498	2001	202
WRKY85	-	LOC105037502	minus	707	1876	232
WRKY86	-	LOC105036260	minus	30951	33207	133
WRKY87	-	LOC105032259	plus	1118523	1122926	251
WRKY88	-	LOC105060191	plus	1154550	1156525	316
WRKY89	-	LOC105060181	minus	776067	784882	535
WRKY90	-	LOC105061299	minus	1132053	1196544	709
WRKY91	-	LOC105032540	minus	1056488	1082285	672
WRKY92	-	LOC105033674	plus	298091	311193	551
WRKY93	-	LOC105034695	plus	157052	174713	545
WRKY94	-	LOC105034934	minus	231238	253849	641
WRKY95	-	LOC105035205	plus	245494	250181	478

### Chromosomal locations, gene structures, and gene duplication of *EgWRKY*

**Fig 1:**
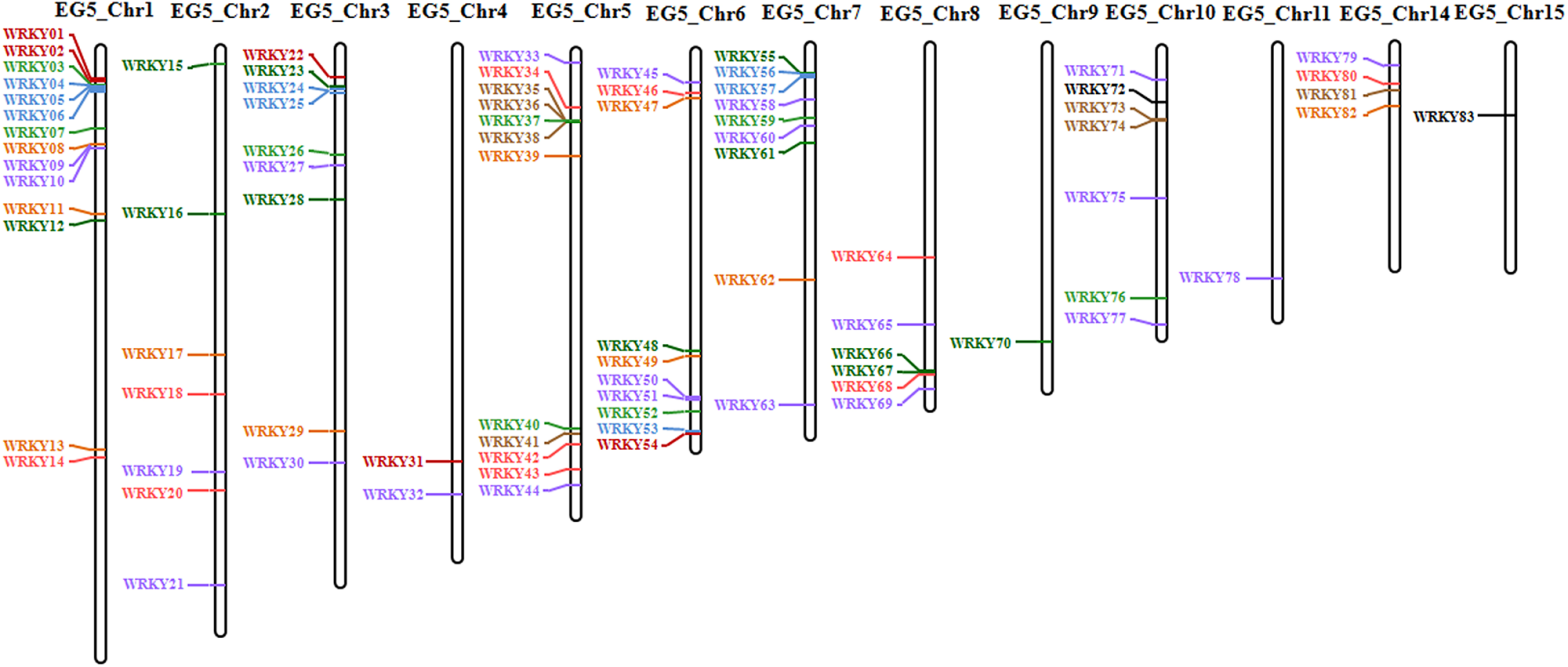
Distribution of *EgWRKY* genes across the chromosomes of *Elaeis guineensis*. Vertical bars represent chromosomes of *Elaeis guineensis*. The chromosome number is indicated at the top of each chromosome. The coloured lines indicate different clusters based on multiple alignments between conserved WRKY domains from the 95 *EgWRKY* genes identified in this study. Dark red: cluster I; Green: cluster II; Blue: cluster III; Yellow: cluster IV; Red: cluster V; Black: cluster VI; Purple: cluster VII; Brown: cluster VIII; Bright green: cluster IX.

The distribution of *EgWRKY* in African oil palm was uneven: 83*EgWRKY* genes were unevenly distributed among13 chromosomes; while no *EgWRKY* genes were detected in chromosomes12, 13 and 16 (Fig 1). Chromosome 1 harboured 14*EgWRKY* genes, the largest number of any chromosome. Moreover, 12*EgWRKY*genes were located on undetermined chromosomes.

**Fig 2:**
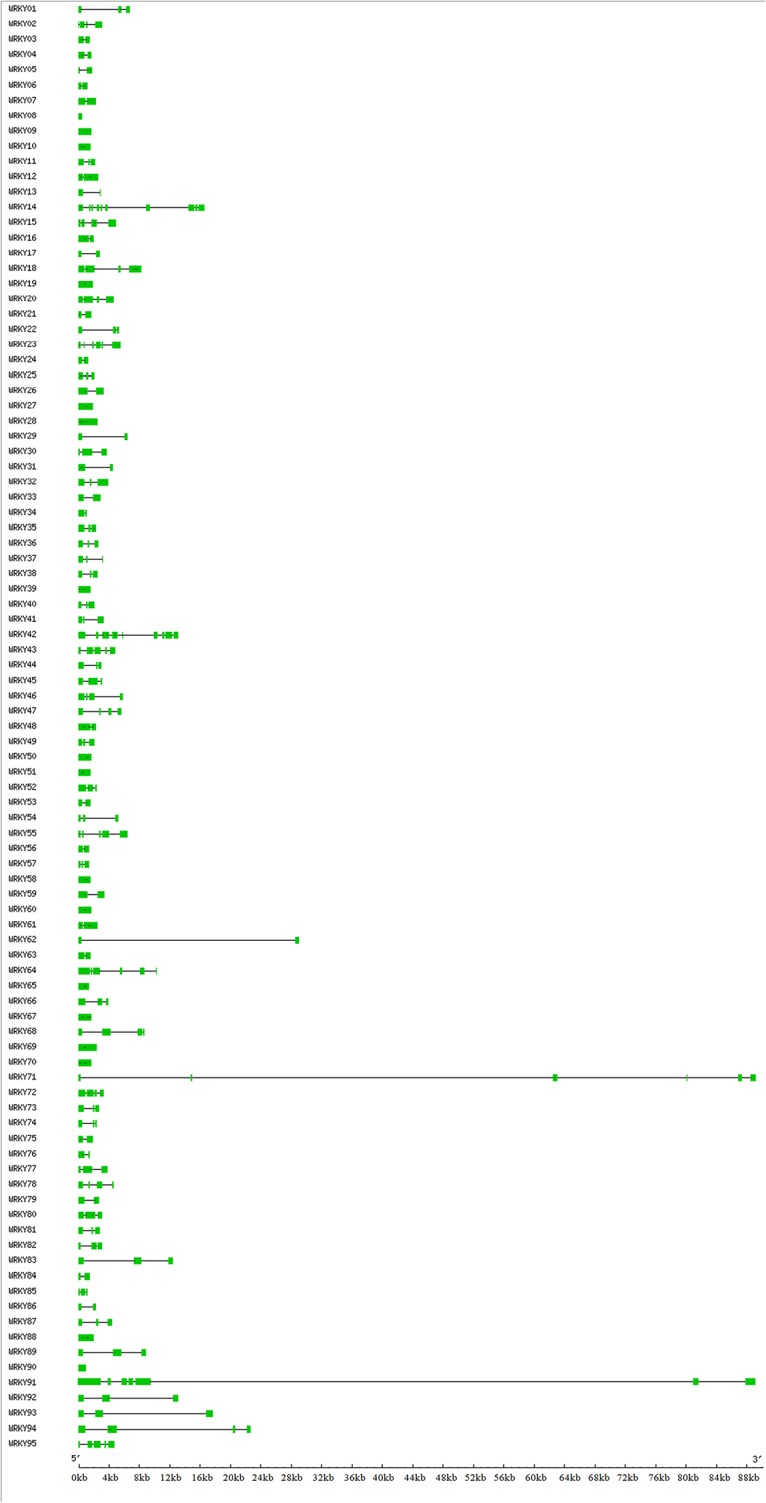
Gene structures of the 95 *EgWRKY* genes identified in this study. Exons are represented by coloured boxes and introns are represented by black lines.

Analysis of *EgWRKY* gene structures indicated a notably variation exist in these genes (Fig 2). The intron numbers of *EgWRKY* genes identified in this study varied from 0 to 12, with an average of 2.99 introns per *EgWRKY* (S1 Table). The largest fraction of *EgWRKY*s (42, 44.21%) contained two introns, followed by *EgWRKY*s containing three introns (20, 21.05%). Large gene size variation were detected in *EgWRKY* which varied from 477 bp (*EgWRKY*08) to 89,167 bp (*EgWRKY*71), with an average size of 5992 bp. The large variations in structure in the members of the *EgWRKY* gene family suggest that the genome of *Elaeis guineensis* has undergone significant divergence during a long evolutionary history.

**Fig 3:**
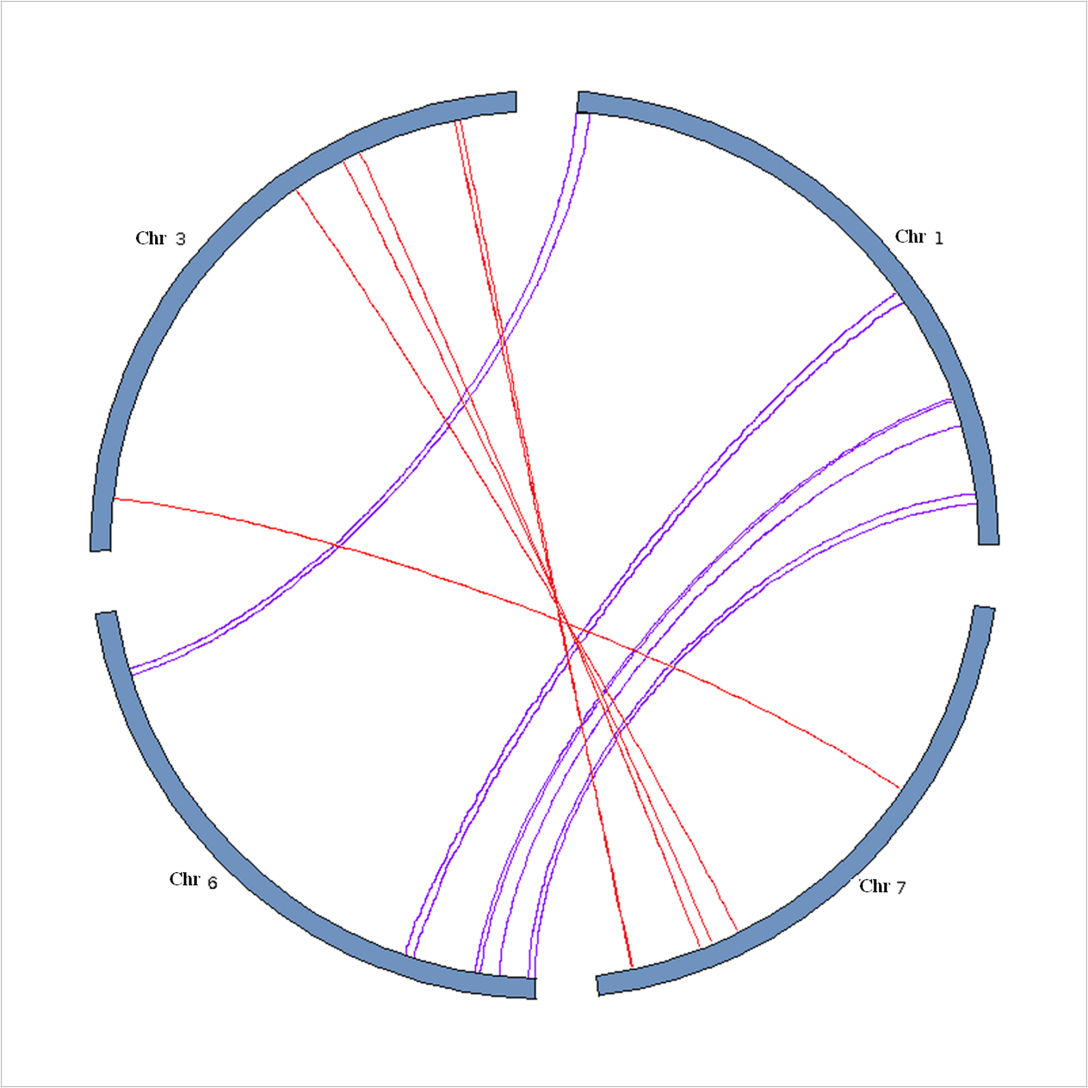
Duplicated genes pairs of *EgWRKY*s identified in this study. The coloured bars represent chromosomes of E*laeis guineensis*.

The segmental duplication in *Elaeis guineensis* defined this species may have originated from a palaeotetrapolyploid. In this study, we investigated the segmental duplication event shappened in blocks containing *EgWRKY*s genes. The gene distribution in oil palm homologous blocks showed that 32 *EgWRKY*s were produced by segmental duplication, and two *EgWRKY*s were produced by tandem duplication. According to whole-genome duplication analysis of *Elaeis guineensis*, four large synteny blocks were detected in chromosomes 1, 3, 6, and 7. The 32 *EgWRKYs* produced by segmental duplication were also found to be located in the four large synteny blocks indicated by Singh et al [1] (Fig 3). Nine pairs of *EgWRKY*s in chromosomes 1 and 6 showed good collinear relationships. Seven other pairs of *EgWRKY*s produced by segmental duplication were detected in chromosomes 3 and 7.

### Classification of Eg*WRKY*s according to conserved WRKY domains

**Fig 4:**
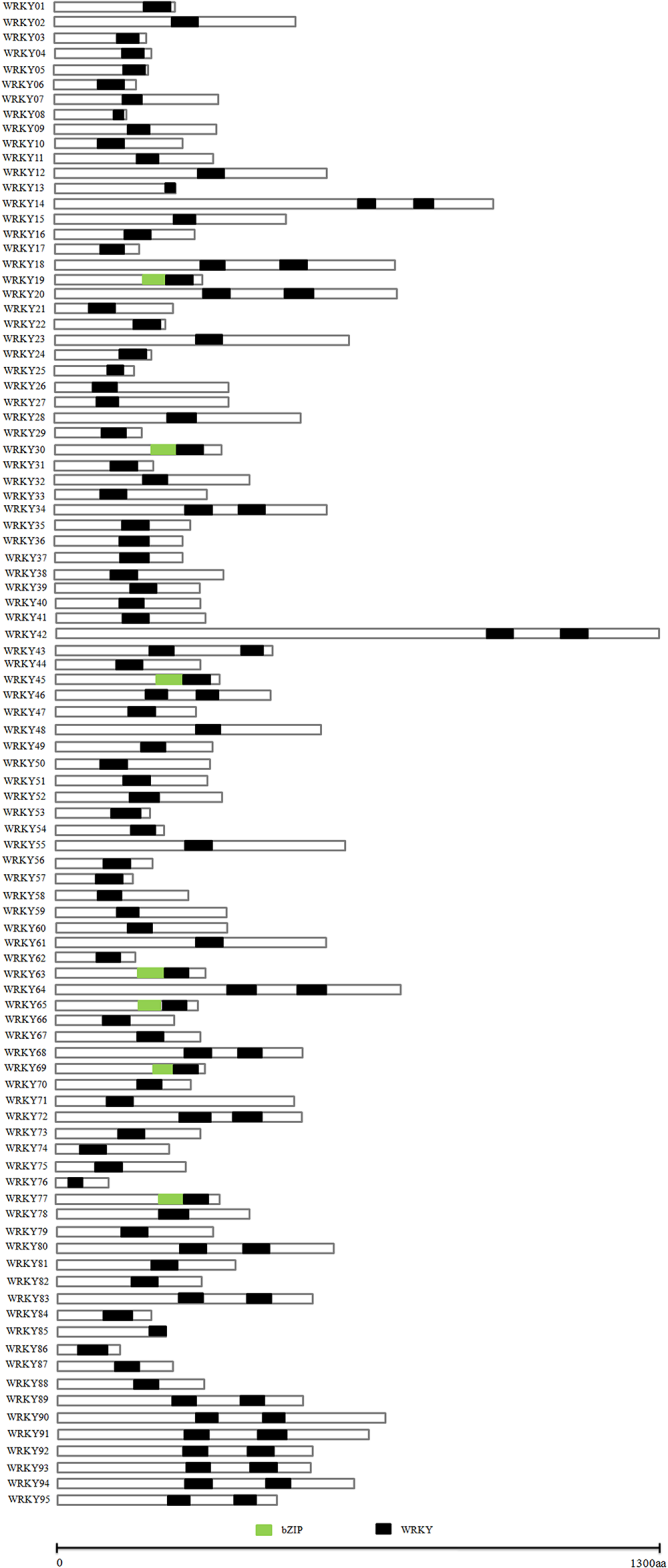
Schematic representation of conserved motifs in 95 *EgWRKY* genes. All motifs were identified by MEME with the full-length amino acid sequence of 95 *EgWRKY* in *Elaeis guineensis*. The size of each *EgWRKY* were displayed proportionally. WRKY and bZIP motif were indicated using different colour box.

**Fig 5:**
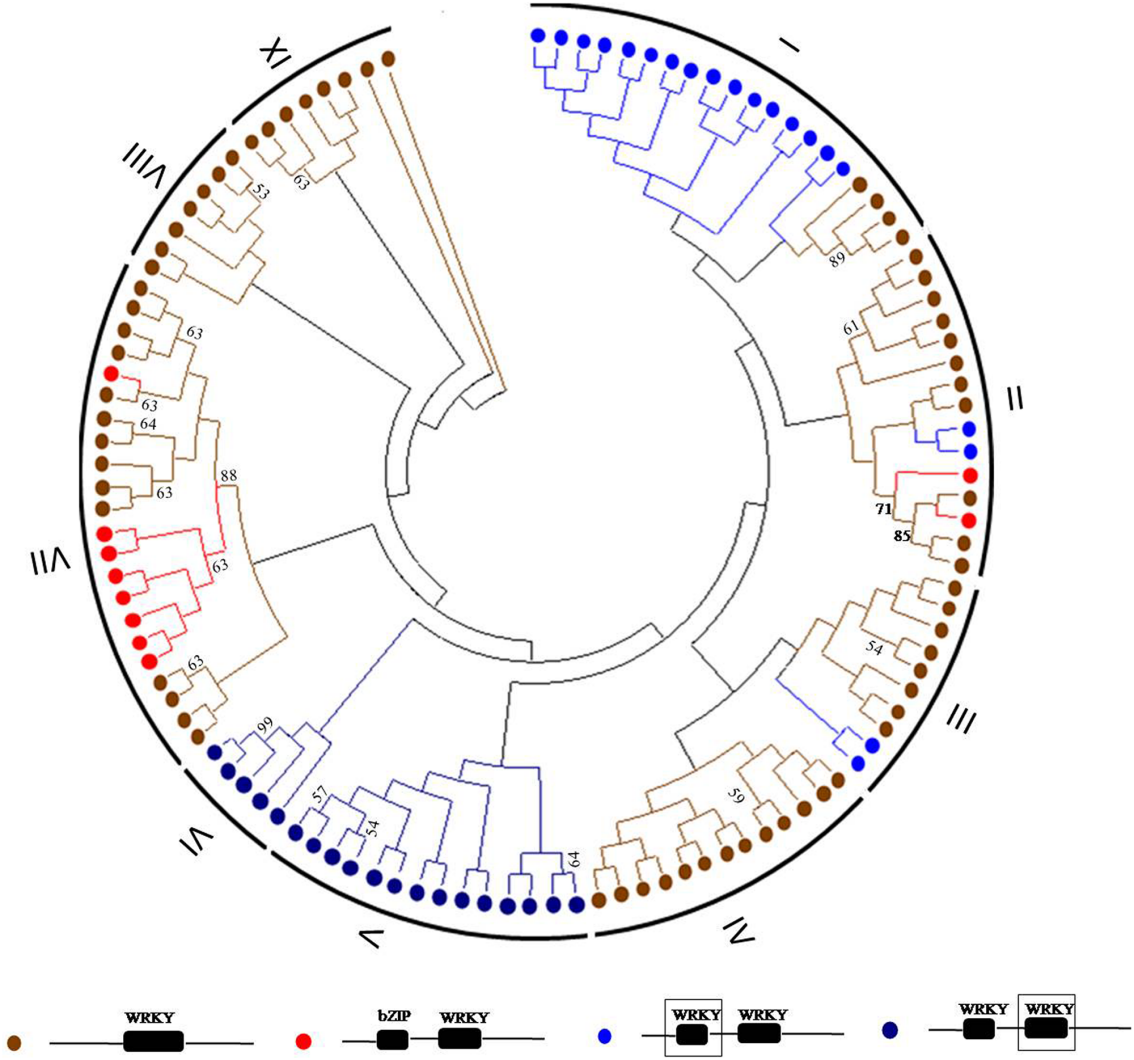
Phylogenetic tree obtained from the neighbour-joining analysis of conserved WRKY motifs from 95 *EgWRKY* genes. *EgWRKYs* that contain only one WRKY domain are marked with brown circles; *EgWRKYs* that contain conserved WRKY and bZIP domains are marked with red circles; *EgWRKYs* that contain two conserved WRKY domains are marked with blue circles.

Of the 95 *EgWRKY*s identified, the majority(67, 70.52%)contained only one conserved *WRKY* domain, 19 (20%) contained two conserved *WRKY* domains, and the remaining 9 (9.48%)contained one conserved C_2_H_2_-type zinc finger domain and one conserved *WRKY* domain (Fig 4). Phylogenetic analyses were performed to evaluate the relationship between the identified *EgWRKYs* by the neighbour-joining analysis. The phylogenetic tree was constructed based on the *WRKY* motif, and 95 *EgWRKY* genes were classified into eight groups (Fig 5). The 67 *EgWRKY*s that contained only one *WRKY* domain clustered into all groups except V and VI. The N-terminal *WRKY* domains in the19 *EgWRKY*s containing two conserved *WRKY* domains clustered into group I, except for *EgWRKY*92, *EgWRKY*93, *EgWRKY*67, and *EgWRKY*70, while the other C-terminal *WRKY* domains were clustered exclusively into groups V and VI. Meanwhile, the *EgWRKY*s containing one conserved C_2_H_2_-type zinc finger domain and one conserved *WRKY* domain were clustered into groupVII, except for *EgWRKY*09, *EgWRKY*37, *EgWRKY*38, and *EgWRKY*85.

### Expression levels of *EgWRKY*s in different tissues based on transcriptome data

**Fig 6:**
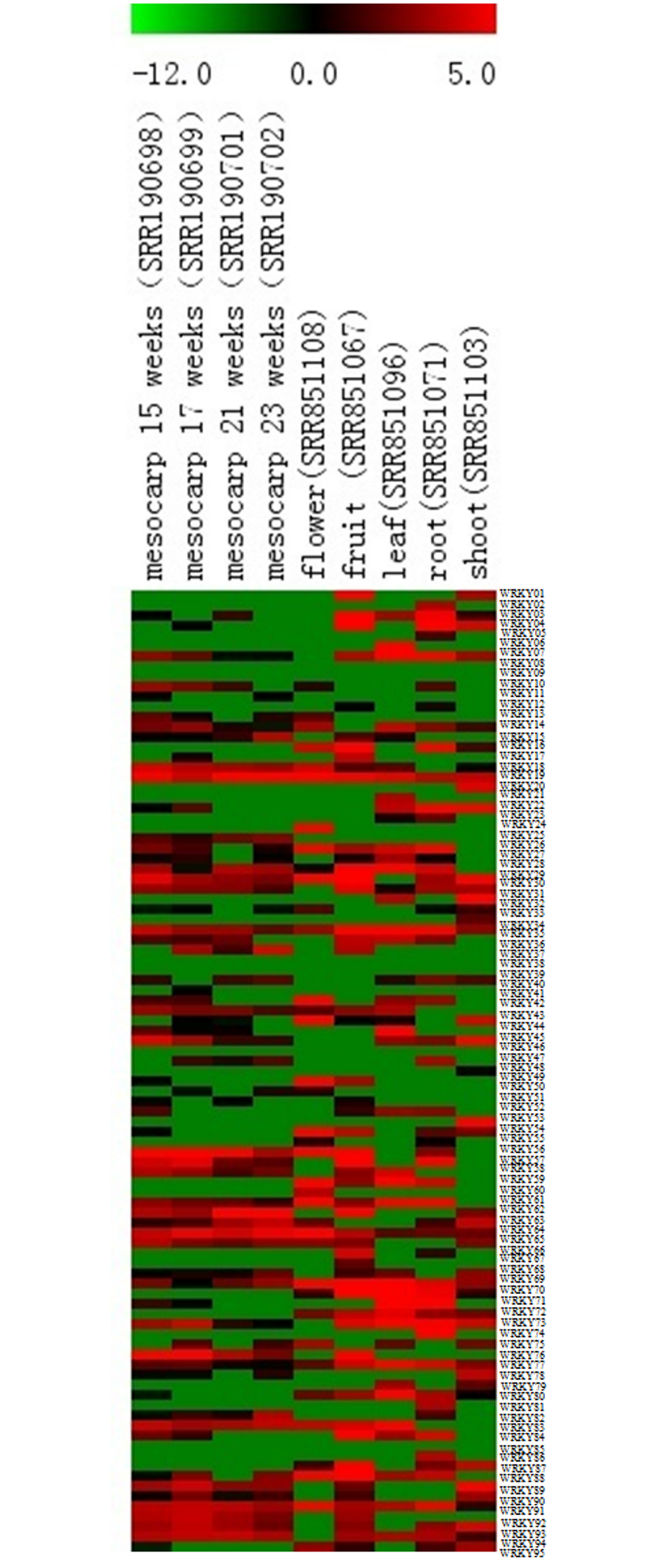
Heat map of *EgWRKY* gene expression in different tissues of *Elaeis guineensis*. Log _2_^RPKM^ values were used to construct the heat map with clustering.

To assess *EgWRKY* expression levels in different tissues of *Elaeis guineensis*, nine transcriptomes were downloaded from the SRA database of NCBI. These transcriptomes covered six oil palm tissues, including mesocarp (five different developmental stages), leaf, fruit, flower, root and shoot tissue. Almost every *EgWRKY* gene expressed (RPKM value > 0) in at least one transcriptome data set, except for *EgWRKY*08, *EgWRKY*09, *EgWRKY*37, *EgWRKY*38, *EgWRKY*40, *EgWRKY*46and *EgWRKY*85 (Fig 6). However, a large proportion of *EgWRKY*s showed low expression levels in the nine transcriptome data sets (RPKM values< 15). *EgWRKY*19had high expression levels (RPKM value > 15) in all five tissues of *Elaeis guineensis*. Three *EgWRKY*s had high expression levels (RPKM value >15) in four tissues (including *EgWRKY*69, *EgWRKY*07 and *EgWRKY*19). Thirty *EgWRKY*s showed high expression levels (RPKM value >15) in only one tissue (including *EgWRKY*01, 02, 05, 06, 11, 20, 21, 24, 25, 30, 31, 33, 35, 36, 41, 44, 48, 49, 51, 53, 58, 60, 64, 66, 67, 74, 80, 81, 93 and94), which indicated that a large proportion of *EgWRKY*s showed a tissue-specific expression pattern. Twenty-nine *EgWRKYs* had low (RPKM value < 15)or no expression in the five tissues of *Elaeis guineensis* (including *EgWRKY*08, 09, 10, 12, 13, 15, 17, 23, 32, 37, 38, 39, 40, 42, 46, 47, 50, 52, 63, 65, 68, 75, 78, 79, 82, 90, 92 and95).

**Fig 7:**
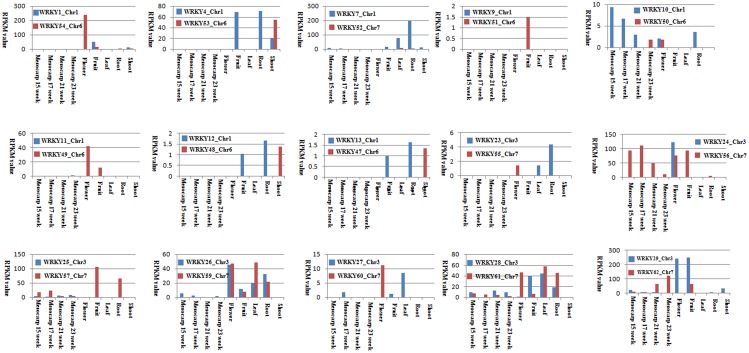
The expression patterns of duplicate genes in different tissues.

Meanwhile, we also investigated the expression patterns of the duplicated gene pairs in different tissues. As shown in Fig 7, the expression levels of most duplicated gene pairs showed different patterns across different tissues, except for *WRKY*24_Chr3/*WRKY*56_Chr7 in flower tissue and*WRKY*26_Chr3/*WRKY*59_Chr7 in flower and root tissue. These two duplicated gene pairs both showed high expression levels in flower and root tissue. These results suggested that subfunctional processes have conducted in these duplicate gene pairs of *EgWRKY* during a long evolutionary history.

### Expression changes of *EgWRKY*s under cold stress

Analysis of the expression level change of *EgWRKY*s under cold stress were conducted based on two transcriptomes: one from a control sample and one from a mixture of samples collected at different times after cold treatment. The RPKM values of all 95 *EgWRKY*s were increased in the trascriptome under cold stress. Additionally, the expression changes of 17 *EgWRKY*s were greater than two-fold under cold stress; these included *EgWRKY*03, 06, 07, 11, 16, 25, 26, 28, 29, 35, 52, 59, 61, 72, 76, 80 and 88.

### Validation of expression of some *EgWRKY*s under cold, drought and salt stress

**Fig 8:**
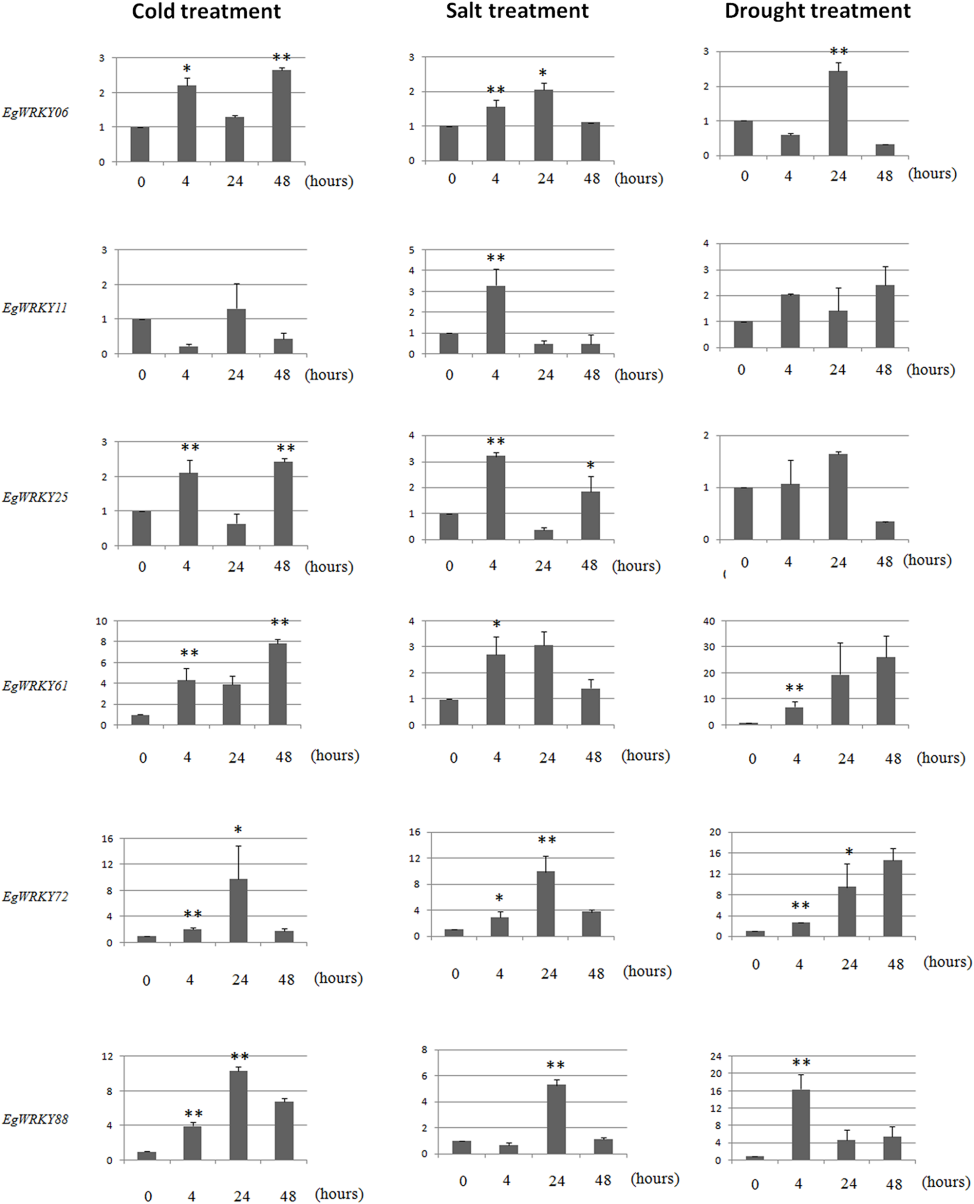
Real-time quantitative PCR validation of *EgWRKY* gene expression under salt, drought and cold treatment. One-way ANOVA was used to test significant difference of expression level between adjacent time periods. * indicated “*p*< 0.05” and ** indicated “*p*< 0.01”.

Based on trasncriptome data under cold treatment, 17 *EgWRKY* genes had increased expression levels at least two fold, six (*EgWRKY*06, 11, 25, 61, 72, and 88) of which were validated using real-time qRT-PCR. As was observed in the transcriptome data under cold stress, the expression of the six *EgWRKY*s could be induced under cold stress (Fig 8) which in accordance with the result from transcriptome data. Meanwhile, these six *EgWRKY*s could also be induced under drought and salt treatment. These data seem to indicate that these six *EgWRKY*s were associated with a wide range of abiotic stress responses in *Elaeis guineensis*.

## Discussion

African oil palm (*Elaeis guineensis*, 2n = 32) is an important tropical oil crop and has high tolerance to drought and salt stress. Currently, African oil palm is mainly cultivated in the tropics with a minimal growth temperature of 15°C and the cultivation area is limited. Low temperature causes cold damage such as yellowing and withering of young leaves and flowers in oil palm. To provide clues for molecular basis of oil palm stress response, the WRKY transcript factor family, which plays a critical role in the regulation of plant stress tolerance, was systematically analyzed in this study. A total of 95 *EgWRKY* genes were identified in African oil palm genome and characterized in four aspects—gene structure variation (477 bp in *EgWRKY*08 to 89,167 bp in *EgWRKY*71), phylogenetic relationship, gene expansion and expression profiles.

According to the amino acid sequences of the conserved domains, phylogenetic and evolutionary relationships among the 95 *EgWRKY*s were established. Previous studies had shown that *WRKY* transcription factors can be grouped into three clusters. Cluster I proteins contain two conserved *WRKY* motifs with a C_2_H_2_ motif. Clusters 2 and 3 are characterized by a single conserved *WRKY* motif[26]. A few studies also showed that in some species, the *WRKY* family may include cluster IV, which contains an incomplete *WRKY* domain [41]. In this study, 95 Eg*WRKY*s were classified into nine clusters. For the N- and C-terminal *WRKY* conserved domains, the phylogenetic analysis showed that they were classified into different classes: N-terminal conserved domains were grouped into cluster I, and C-terminal domains were grouped into clusters V and VI. *WRKY* genes with conserved *WRKY* and bZIP motifs were grouped into cluster VII. *WRKY*s with single conserved motif were mainly grouped into clusters II, III, IV, VIII, and IX. *EgWRKY*13, *EgWRKY*40, and *EgWRKY*74, which were found to contain incomplete conserved motifs, were grouped into clusters II, VIII, and IX, respectively.

Gene structure diversity was thought to be able to reflect the evolutionary relationships of gene families, providing additional information for phylogenetic classification [42]. In this study, the numbers of introns found in the identified *EgWRKY* genes varied from 0 to 12, with an average of 2.99 introns per *EgWRKY*. Some previous studies have shown the diversity of gene structure in various species. The number of introns of cassava varied from 1 to 5[34]. In rice and rubber tree, the number of introns varied from 0 to 8 and 1 to 7, respectively[43, 44]. These results indicated that oil palm had more gene structure diversity than did cassava, rice and rubber tree. Meanwhile, the largest fraction of *EgWRKY*s (42, 44.21%) contained two introns. A similar phenomenon was also detected in cassava (42 of 85 *MeWRKY* genes contained two introns), rice (42 of 92) and rubber tree (40 of 81). Cluster I members contained 1 to 12 introns; cluster II, 3 to 6; cluster III, 2 to 4; cluster IV, 0 to 3; cluster V, 1 to 12; cluster VI, 0 to 6; cluster VII, 2 to 3 with the exception of*EgWRKY*71; and clustersVIIIandIX, 2 to 3. Previous research showed that intron loss occurs more rapidly than intron gain after segmental duplication in rice. Consequently, some *EgWRKY*s from clusters I and V may be original genes compared to other *EgWRKY*s in *Elaeis guineensis*.

Some previous studies showed that the expression of *WRKY* transcription factors can be induced by abiotic stress and play crucial roles in regulating plant responses to abiotic stress [8–14]. In Arabidopsis, *WRKY*34 was found to be involved in cold stress and is a negative regulator of cold response[45]. *AtWRKY*34 showed high similarity to*EgWRKY*18 and*EgWRKY*64. Based on transcriptomic data, expression of *EgWRKY*18 and*EgWRKY*64 can be induced under cold stress. In Arabidopsis, *AtWRKY*30expression can be induced by abiotic stress and MV, H_2_O_2_, arsenic, drought, NaCl, and mannitol treatment. Overexpressing *AtWRKY*30can significantly increase the tolerance of Arabidopsis to MV and salinity stress[14]. *AtWRKY*30 showed high similarity to *EgWRKY*07 and *EgWRKY*52. According to transcriptome data, expression of these two *EgWRKY*s was strongly induced, up-regulated 3.367- and 11.883-fold, respectively. The C-terminal domain of *AtWRKY*33 can interact with multiple VQ proteins to regulate multiple abiotic stresses [10]. *AtWRKY*33has high similarityto*EgWRKY*34and *EgWRKY*42, and these two *EgWRKY*s were up-regulated 2.72- and 1.15-fold under cold treatment, respectively. Based on transcriptome data and qPCR results, the expression of almost all the *EgWRKY*s was induced under abiotic stresses. This indicated that *EgWRKY*s may play important roles in oil palm responses to abiotic stresses.

## Supporting information

S1 Text Gene sequences of 95 *EgWRKY*s identified in the study. (TXT)

S2 Text Amino acid sequence of 95 *EgWRKY*s identified in the study. (TXT)

S1 Table Exon position of every *EgWRKY* gene in the chromosome of *Elaeis guineensis*. (XLSX)
